# Supplementary figures and images for: Countering the advert effects of lung cancer on the anticancer potential of dendritic cell populations reinstates sensitivity to anti-PD-1 therapy
Source: PLoS One. 2021 Nov 30;16(11):e0260636. doi: 10.1371/journal.pone.0260636 (PMC8631683; doi:10.1371/journal.pone.0260636)

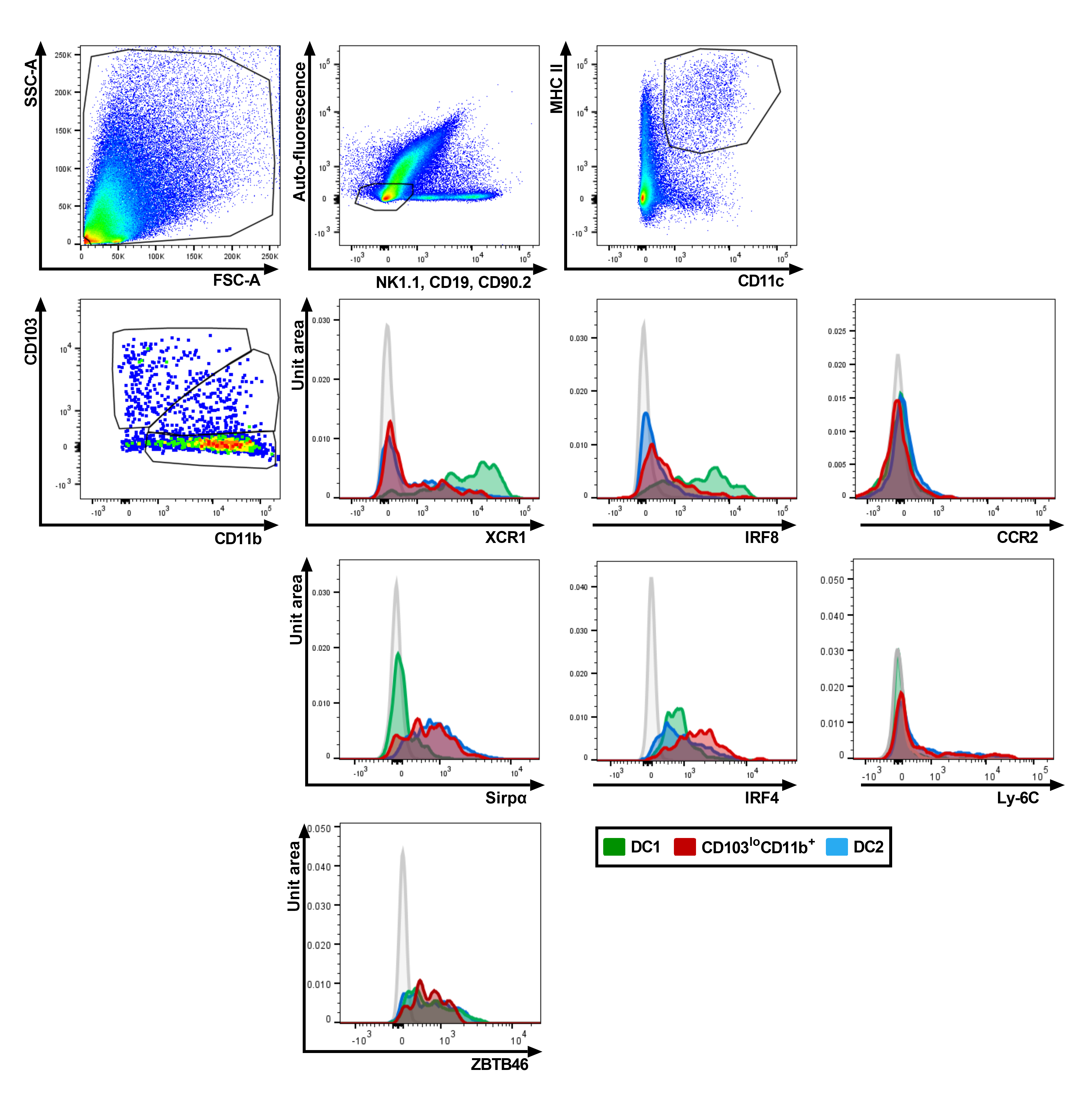

Supplement: S1 Fig — Gating strategy for the identification of DCs, associated with Fig 4. Total DCs were gated on auto‐fluorescence-, NK1.1-, CD90.2-, CD19-, MHC IIHi and CD11c+. For this Fig, three populations were segregated prior to the further analysis of DC1 and DC2 markers, i,e CD103+CD11b-/lo (green), CD11b+CD103- (blue) and CD103loCD11b+ DCs (red). A representative flow cytometry histogram showing the normalized number of cells (unit area) on the Y-axis and fluorescence intensity on the X-axis is presented for the XCR1, Sirpα, IRF8, IRF4, CCR2, Ly-6C and ZBTB46 expression for each of these three DC populations. FMO controls appear in grey in each histogram. (TIF) [file pone.0260636.s001.tif]

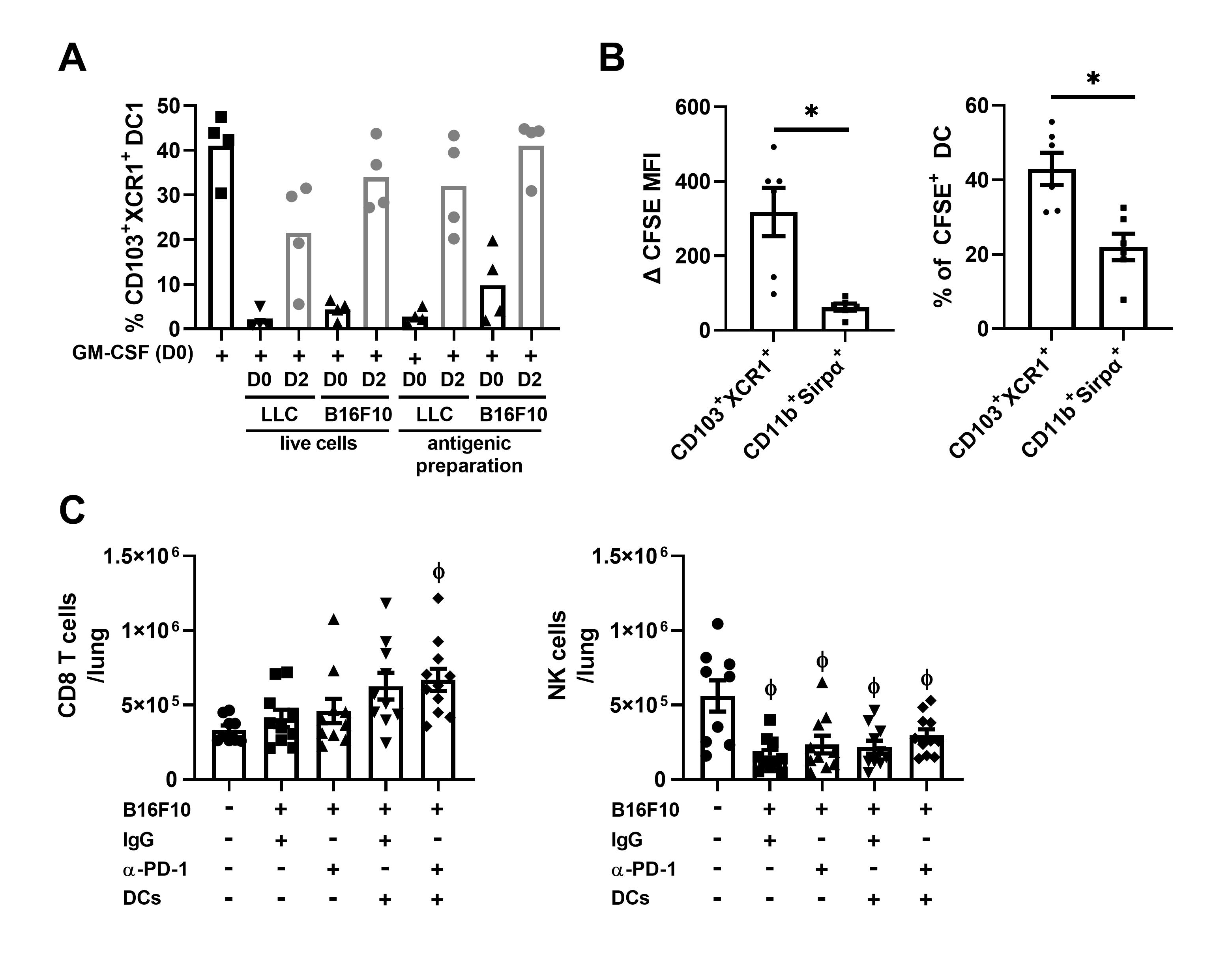

Supplement: S2 Fig — (A) FLT3L-BMDCs were stimulated with GM-CSF (Day 0). Either live B16F10/ LLC cells or a B16F10/ LLC antigenic preparation was added on day 0 or day 2 and the percentage of CD103+XCR1+ DCs was measured by flow cytometry on day 3. Data are presented as individual dots with means. n = 4 pooled from two independent experiments. (B) Two days following GM-CSF stimulation FLT3L-BMDCs were stimulated for 24h with CFSE-treated B16F10 cells and Δ CFSE MFI (CFSE MFI of DC exposed to CFSE-B16F10 –CFSE MFI of unexposed DC (negative control)) in CD103+XCR1+ DC1 and CD11b+Sirpα+DC2 and the percentage of CD103+XCR1+ DC1 and CD11b+Sirpα+DC2 of the total CFSE+ population was measured by flow cytometry. Data are expressed as mean ± SEM. n = 6, pooled from two independent experiments. * = p < 0.05 using a paired t-test. (C) The total number of lung CD8 T cells and NK cells 18 days following B16F10 injections. CD8 T cells were identified as CD45+, CD19-, CD90.2+, CD4-, CD8+ and NK cells were identified as CD19-, CD3e-, B220-, CD49b+, NK1.1+ by flow cytometry analysis. Data are expressed as mean ± SEM. n = 9–11 pooled from two independent experiments. ϕ = p < 0.05 compared to naïve mice. P-values were determined using a one-way ANOVA followed by Tukey’s multiple comparisons test. (TIF) [file pone.0260636.s002.tif]
