# Supplementary material for: Countering the advert effects of lung cancer on the anticancer potential of dendritic cell populations reinstates sensitivity to anti-PD-1 therapy
Source: PLoS One. 2021 Nov 30;16(11):e0260636. doi: 10.1371/journal.pone.0260636 (PMC8631683; doi:10.1371/journal.pone.0260636)
Supplement: Attachment — Submitted filename: RESPONSE TO REVIEWERS.docx [file pone.0260636.s003.docx]

**RESPONSE TO REVIEWERS**

Dear Dr Najbauer,

Here is a point by point response to the minor criticism raised by reviewers. We hope this response, along with the revised manuscript and figures provided here, will be deemed acceptable for publication in PLOS-One. We thank you for the opportunity to review this work,

Marie-Renee Blanchet,

Corresponding author

**Journal requirements:**

1. We have revised the document to meet PLOS one requirements.
2. We have removed all data now shown in the manuscript.
3. Figure 7 does not have copyright. It was done from scratch using the Power Point application by Julyanne Brassard, first author.

**Reviewer 1:**

1. We thank R1 for this pertinent question. All DC populations are increased in cancer, but the proportions of each population are, as reported, very different. Therefore, even if DC1s increase in numbers following cancer development, they are dramatically overtaken by DC2s in this context, which also increase in numbers, but un much higher proportions. Also, anticancer DC1s are found in a 1:1 ratio with regulatory CD103^lo^/CD11b^+^ DCs following tumour development. Taking this into consideration, it becomes clear that the data which speaks the most as to the nature of the DC response in the lung remains DC proportions. We hope this clarifies our reasoning for not including total numbers in figures.
2. This is an interesting question. Quickly, precursors do not express CD103 in the blood, so CD103 low DCs as a biomarker could be used, but would need to come from the lung.
3. We thank R1 for this important question, and have now provided the information in the text.
4. We thank the reviewer for this interesting suggestion and have provided additional information with regards to potential tumour molecules interactions in the text.

**Reviewer 2:**

General: we appreciate R2’s feedback on the quality of grammar in the text, and have consequently carefully reviewed the text.

Introduction: We have added information of PD-L1 therapy as proposed by the reviewer.

Methods: we thank R2 for this useful suggestion and have provided the information in a separate paragraph as suggested.

Results:

- We agree with R2 that performing functional assays on CD103lo CD11b DCs would be interesting. We have considered this often, and are unfortunately limited by the low total number of DCs which can be isolated from lung. We have evaluated that we would need over 10 mice per condition to have enough cells to perform functional assays, which, ethically, is not acceptable for our local committee. We therefore try to circumvent this technical limitation by adjusting our message and insuring that we discuss the regulatory “potential” of this population rather than affirming they possess a regulatory function. We hope this explanation will satisfy the reviewer but understand their feedback. Of note, many publications seem limited by this technical limitation with regards to regulatory DCs, and publish regulatory molecule expression as a marker of DC regulatory potential.
- We thank R2 for the feedback on consistency in figure naming and have adjusted the text accordingly.
- The caption was corrected as suggested by R2.
